# Wtip- and Gadd45a-Interacting Protein Dendrin Is Not Crucial for the Development or Maintenance of the Glomerular Filtration Barrier

**DOI:** 10.1371/journal.pone.0083133

**Published:** 2013-12-20

**Authors:** Zhijie Xiao, Patricia Q. Rodriguez, Liqun He, Christer Betsholtz, Karl Tryggvason, Jaakko Patrakka

**Affiliations:** 1 Division of Matrix Biology, Department of Medical Biochemistry and Biophysics, Karolinska Institutet, Stockholm, Sweden; 2 Division of Vascular Biology, Department of Medical Biochemistry and Biophysics, Karolinska Institutet, Stockholm, Sweden; 3 Department of Immunology, Genetic and Pathology, Uppsala University, Uppsala, Sweden; 4 Cardiovascular and Metabolic Disorders Program, Duke-NUS, Singapore; Fondazione IRCCS Ospedale Maggiore Policlinico & Fondazione D'Amico per la Ricerca sulle Malattie Renali, Italy

## Abstract

Glomerular podocyte cells are critical for the function of the renal ultrafiltration barrier. Especially, the highly specialized cell–cell junction of podocytes, the slit diaphragm, has a central role in the filtration barrier. This is highlighted by the fact that mutations in molecular components of the slit diaphragm, including nephrin and Cd2-associated protein (Cd2ap), result in proteinuric diseases in man. Dendrin is a poorly characterized cytosolic component of the slit diaphragm in where it interacts with nephrin and Cd2ap. Dendrin is highly specific for the podocyte slit diaphragm, suggesting that it has a dedicated role in the glomerular filtration barrier. In this study, we have generated a dendrin knockout mouse line and explored the molecular interactions of dendrin. Dendrin-deficient mice were viable, fertile, and had a normal life span. Morphologically, the glomerulogenesis proceeded normally and adult dendrin-deficient mice showed normal glomerular histology. No significant proteinuria was observed. Following glomerular injury, lack of dendrin did not affect the severity of the damage or the recovery process. Yeast two-hybrid screen and co-immunoprecipitation experiments showed that dendrin binds to Wt1-interacting protein (Wtip) and growth arrest and DNA-damage-inducible 45 alpha (Gadd45a). Wtip and Gadd45a mediate gene transcription in the nucleus, suggesting that dendrin may have similar functions in podocytes. In line with this, we observed the relocation of dendrin to nucleus in adriamycin nephropathy model. Our results indicate that dendrin is dispensable for the function of the normal glomerular filtration barrier and that dendrin interacts with Wtip and Gadd45a.

## Introduction

The renal ultrafiltration occurs through the capillary wall of the kidney glomerulus. The filtration barrier is formed of fenestrated endothelial cells on the inside, the glomerular basement membrane in the middle, and specialized glomerular epithelial cells (podocytes) on the outside. Although all three layers have important roles in the filtration barrier function, the podocyte seems to be the most critical part of the renal filter. This is underlined by the fact that mutations in many podocyte proteins cause albumin leakage through the glomerular barrier [1]. This protein leakage is clinically observed as proteinuria. Proteinuria is a common symptom of renal disorders and considered to be a major factor in promoting the progression of renal diseases. Therefore, it is of critical importance to understand the biology of the glomerulus filtration barrier.

Podocytes can morphologically be divided to a cell body and cytoplasmic extensions termed major and foot processes [2]–[3]. Foot processes enwrap the glomerular capillaries and adjacent foot processes are interconnected by highly specialized cell-cell junctions called slit diaphragms. The slit diaphragm is formed of a complex of transmembrane proteins. Many of these proteins, such as nephrin and podocin, are highly specific for the podocyte cell and essential for the maintenance of the filtration barrier [4]. Importantly, mutations in these proteins are responsible for many inherited proteinuric syndromes in man [1]. At the cytoplasmic side, the slit diaphragm is connected to the actin cytoskeleton via a number of linker proteins, including Nck and Cd2-associated protein (Cd2ap). Studies in knockout mice have shown that also these linker proteins are crucial for the maintenance of the kidney filter [5]–[6].

Dendrin is a poorly characterized cytoplasmic protein that was originally identified to be present in dendritic processes of neurons [7]. We have identified dendrin as a component of the podocyte slit diaphragm [8]–[9]. Dendrin is highly specific for the podocyte cell as RNA expression was detected only in podocytes and neuronal tissue. Furthermore, Asanuma et al. showed that dendrin interacts with Cd2ap and nephrin in the slit diaphragm, and that in injured podocytes dendrin can relocate to nucleus and modulate TGF-B-mediated apoptotic signals [10]. In addition, in human patients with IgA nephropathy, dendrin has been reported to trasnlocate to nucleus [11], suggesting that dendrin has a role in the pathogenesis of this common renal disorder. However, the functional role of this highly podocyte-specific gene in the kidney filter is still unknown.

In this study, in order to enlight the role of dendrin in the glomerulus filtration barrier, we have generated a dendrin knockout mouse line, as well as performed protein-protein interaction studies. Our results indicate that dendrin binds to Wt1-interacting protein (Wtip) and growth arrest and DNA-damage-inducible 45 alpha (Gadd45a), two proteins that are known to be present in the nucleus and mediate transcription. Furthermore, our studies in dendrin-deficient mouse line indicate that dendrin is not needed for the development or function of the glomerulus filtration barrier.

## Materials and Methods

All animal studies were carried out in Scheelelaboratoriet (Karolinska Insitutet) and were approved by the Committee on Research Animal Care (Stockholms Norra djurförsöksetiska nämnd).

### Generation of dendrin-deficient mouse line

Dendrin knockout mice were generated using the Velocigene™ technology (Regeneron Pharmaceuticals, Inc.) [12]. The coding sequence of exons one and two, which represent the whole coding sequence of dendrin, were replaced with an eGFP-containing cassette (eGFP lox-Ub1- EM7- Neo- lox cassette). Thus, the genomic sequence of dendrin (position 99265856–99268375 bp in chromosome 15) from start codon ATG to the stop codon TGA was replaced, in frame with respect to the dendrin initiation codon, by the coding sequence of eGFP and a Lox flanked neomycin gene driven by a mammalian promoter. Correctly targeted ES cells (derived from the 129S6SvEv/C57BL6 mouse strain) were identified using the loss-of-negative-allele assay (Valenzuela et al., 2003), and the proper integration of the knockout cassette was verified by sequencing of the integration sites (both the 5′ and 3′ junctions). Correctly targeted ES line was used to generate chimeric male mice that were then bred to C57BL/6 female to generate N1 mice. Heterozygous mice were mated with each other to obtain wild-type, heterozygous, and null mutant mice. The mice used in this study were backcrossed over 5 generations (N5 to N7) onto the C57BL/6 background.

### Genotyping

Genotyping was done by PCR using genomic DNA extracted from ear biopsies. For the detection of the wild-type allele, the primer pair ‘WTL’ (5′- GGAGGATCTCAGCGTCCATA -3′ in Exon 2) and ‘WTR’ (5′- AGGTTCAAGGCCTCTCCATT -3′ in three prime untranslated region) generating a 574 bp band was used. The null allele was detected with primer ‘KOL’ (5′- AATTCCATCAGACCTCGACCT -3′ in the eGFP-Neo containing cassette) and ‘WTR’ primer generating a 405 bp band. The absence of dendrin mRNA was further confirmed by generating glomerular cDNA from wildtype and knockout mice and by using dendrin-specific primers: exon 1: L 5′-CTGGATGGCCCGTTATTCT-3′, R 5′-CAGTAGCTGGCCTGGATGTC-3′; exon 2: L 5′-GAGCTTGGGGTCTCAGACAG-3′, R 5′-GGTCACCTTCCAAACTTCCA-3′; exon 1–2: L 5′-TTATAGTCGTCGCGCTCCTT-3′, R 5′-CTGATCGCGGACCTAGAGAG-3′. The PCR reaction and analysis was done using standard procedures.

### Immunohistochemistry and histological analysis

For immunofluorescence staining, samples were collected from mouse kidneys, embedded in OCT compound (Sakura, Inc.) and snap-frozen on dry ice. For detecting dendrin, we used three different rabbit polyclonal antibodies. Two of them have been described previously [8], [10], whereas the third was purchased from EDM Millipore. The immunohistochemistry procedure has been described previously [8]. Other antibodies used in this study were anti-Wt1 (Calbiochem), anti-Gadd45α (H-165) (Santa Cruz Biotechnology, inc.), anti-nephrin (Sigma), anti-podocin (Progen), and anti-synaptopodin (Progen). For double staining of dendrin with other rabbit antibodies, the dendrin antiserum was protein A purified and then directly labeled with Alexa Fluor® 488 or 568 (Invitrogen). The labeled dendrin antibody was added secondary to other antibody reaction.

To quantify dendrin positive podocyte nuclei, we double stained dendrin with a known podocyte nucleus marker wt1. Colocalization of dendrin and wt1 (observed as yellow fluorescence) was considered as dendrin expression in a podocyte nuclei. We counted a total number of wt1 positive cells in 10 glomeruli (in each mouse) and correlated this to the number of podocyte nuclei showing dendrin expression.

For histological analysis, kidney samples were fixed in 4% paraformaldehyde followed by dehydration and embedding in paraffin. Sections (3–5 µm) were cut and stained with hematoxylin and eosin.

### Urine analysis

Urine samples were collected from dendrin knockout and control mice for up to 1 year of age. The presence of albuminuria was analyzed by running 2 µl of urine on SDS-PAGE gel (Invitrogen) which was stained with Coomassie blue or PAGE-Blue stain (Fermentas). The stained gels were then scanned and analyzed by Quantity One software (Bio-Rad) to compare albuminuria level between samples.

### Mouse disease models

We induced glomerular injury and proteinuria in dendrin knockout mice by LPS-injection and albumin overload as previously described [13]–[14]. For the LPS-induced nephropathy, we used 8 wild-type and 8 dendrin-null adult mice. Each mouse was injected with 13 µg/g body weight of LPS (0.5 mg/ml diluted in PBS) intraperitoneally. Urine was collected at the time of LPS injection, at 12, 24, 36, 48, 60, and 72 hours after the injection. For the albumin overload experiment, 6 wild-type and 5 knockout adult mice were used. We injected 200 µg of BSA in a volume of 400 µl intraperitoneally on four consecutive days. Urine samples were collected before each injection and 24 hours after the last injection. Urine samples were analyzed as described above. The data was analyzed by pair wised t-test. P>0.05 was considered as a significant difference.

Adriamycin induced proteinuria model was established in BALB/c mouse strain, which has been shown to be susceptible to this toxin [15]. Adriamycin (Sigma) was diluted with isotonic saline solution (0.9% NaCl_2_) to 2 mg/mL, and singly injected via the tail vain at a dosage of 10.5 mg/kg body weight.

### Yeast two-hybrid assay

The coding sequence of mouse dendrin was cloned to a bait plasmid pGBKT7, and used to screen a custom-generated mouse kidney glomerulus cDNA library [16]. The yeast two-hybrid (y2h) screening was performed according to the manufacturer's instructions (Clontech Laboratories). The positive clones were sequenced and analyzed with NCBI's BLAST database.

### Western blotting and co-immunoprecipitations

Western blotting was performed using standard procedures. Anti-β-actin antibody (Abcam) was used as a loading control. For coimmunoprecipitations, HEK293 cells were co-transfected with full length myc-tagged Gadd45a and flag-tagged dendrin constructs, or full length myc-tagged Wtip and ha-tagged dendrin constructs, respectively. We used irrelevant expression constructs as controls (Myc-STN or PSL). Experiments were performed according to standard procedures.

### Microarray analysis

Three dendrin-null and three littermate mice at age of 13 months were used to profile glomerular transcriptomes. Glomeruli were isolated as described previously (Takemoto et al., 2002) and total glomerular RNA was extracted using RNeasy® Mini Kit (Qiagen). Microarrays were performed using Mouse Genome 430 2.0 Array according to standard procedures as described by the manufacturer (Affymetrix, Santa Clara, Calif., USA).

Affymetrix raw data were normalized using Bioconductor GCRMA package (Bioconductor gcrma package, version 2.30.0), and Significance Analysis of Microarrays was performed to identify the significantly differentially expressed genes (Bioconductor Siggenes package, version 1.8.0).

## Results

### Establishment of dendrin-deficient mouse line

**Figure 1:**
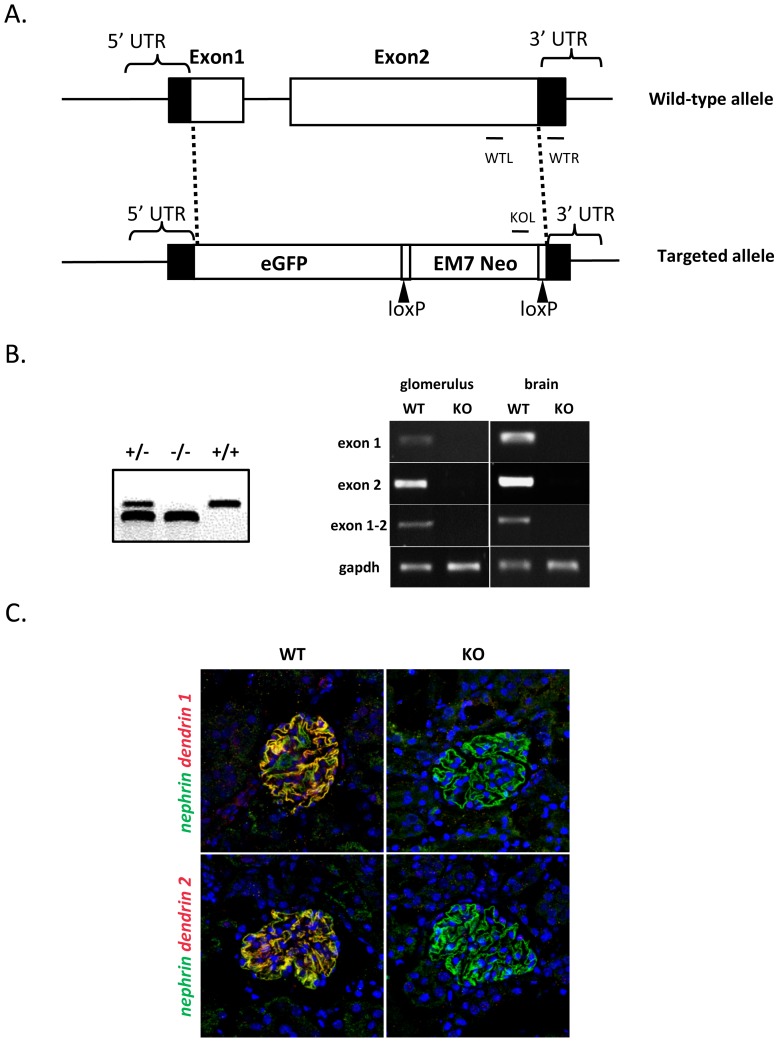
Generation of dendrin knockout mouse line. (a) The targeting construct was designed to replace both coding exons of dendrin with a cassette encoding for eGFP and neomycin-resistance gene. (b) PCR-based genotyping generated 574 bp for a wild-type allele and 409 bp fragment for a knockout allele. The absence of dendrin mRNA was confirmed using RT-PCR in where cDNA generated from wildtype (wt) glomeruli and brain tissue showed dendrin expression (exon 1, exon 2 and the combination of exon 1–2), whereas cDNA from knockout (KO) animals remained negative for dendrin expression. Gapdh was used as a loading control. (c) Double immunofluorescence labeling for dendrin (two different antiboides, dendrin 1 and dendrin 2) and nephrin (green) showed that strong expression in wt glomeruli in where they colocalized (yellow). In KO glomeruli, no signal for dendrin was observed. DAPI staining (blue) showing nuclei. Magnifications: ×200.

Two coding exons of dendrin and the intervening intron were replaced with an eGFP lox-PGK- EM7- Neo- lox cassette (Fig. 1a). Heterozygous mice were mated to obtain dendrin null mice and littermate controls. Pups were born at an expected Mendelian frequency (data not shown). The elimination of dendrin mRNA in homozygous mice was confirmed by RT-PCR analysis and microarray profiling. Dendrin knockout mice lacked the expression of both dendrin exons in glomerulus and brain tissues, whereas control mice showed the presence of these transcripts (Fig. 1b). The absence of dendrin mRNA was further confirmed by our microarray profile that showed approximately 400-times downregulation of dendrin in knockout glomeruli (table S1). The absence of dendrin protein was shown by immunohistochemistry in which two different dendrin antibodies did not detect dendrin in knockout glomeruli, while control glomeruli showed strong immunostaining (Fig. 1c). In Western blot analysis, we somewhat surprisingly found that three different anti-dendrin antibodies recognized a protein around 80 kD in both wildtype and knockout kidney lysates (supporting Fig. S1). In the brain, two of these antibodies recognized similarly a band around 80 and 90 kD in both wildtype and knockout brain tissues. These results correspond to the previously published data by us and others [8], [10]. In addition, these bands were detected in the liver, although no dendrin mRNA has been detected in liver tissues [8]. As our RT-PCR, microarray and immunostaining convincingly indicated that dendrin knockout mice lack dendrin mRNA and protein, we believe that these bands in Western blot represent cross-reactivity against some other protein as all anti-dendrin antibodies used were raised against the C-terminal part of dendrin. The expression of eGFP was analyzed using direct microscopy and immunohistochemistry with anti-GFP antibody. Neither of these approaches detected eGFP signal in the glomerulus (data not shown). Taken together, the results indicate that the mouse line generated is lacking dendrin expression in the glomerulus and the brain.

### Lack of dendrin does not affect glomerular development or barrier function

**Figure 2:**
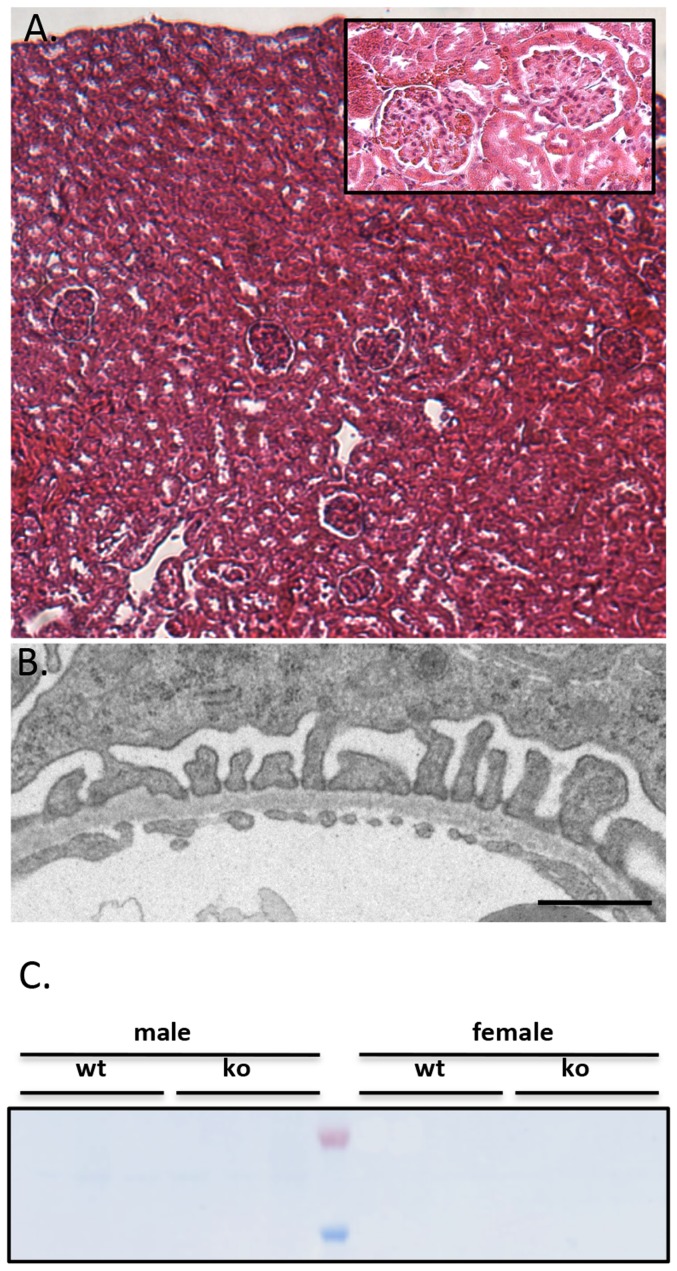
The kidney phenotype in dendrin knockout mouse. (a) Dendrin-deficient mice exhibit normal renal morphology by light microscopic examination (hematoxylin-eosin stained section from 2-month old dendrin knockout mouse). Inset: a dendrin-deficient glomerulus with normal morphological features. (b) In electron microscopy podocytes, the glomerular basement membrane and glomerular endothelial cells show normal morphological features. Fine slender podocyte foot processes interconnected by intact slit diaphragms are observed. Bar = 500 nm. (c) 2 ul of urine from 1-year-old wildtype (wt) and knockout (ko) mice was run on SDS-page gel. No albuminuria is detected Ladder showing 50 kD (blue) and 70 kD (orange) bands.

Previously, we and others have located dendrin specifically to the cytoplasmic side of the podocyte slit diaphragm [8], [10]. As the slit diaphragm is a central player in the glomerular filtration barrier function, we investigated in detail the developing and mature glomeruli in dendrin knockout mice. Light microscopic examination showed that the glomerulogenesis proceeded normally in dendrin knockout mice and mature glomeruli showed normal morphology (data not shown, Fig. 2a). In electron microscopy, dendrin null glomeruli exhibited normal morphology with interdigitating foot processes and intact slit diaphragms (Fig. 2b). Urinanalysis of dendrin knockout mice did not show significant albuminuria (as observed for up to 1 year of age, fig. 2c). Taken together, these results indicate that dendrin is dispensable for the development and maintenance of the glomerular filtration barrier.

### Expressional profiling of dendrin-deficient glomeruli

**Figure 3:**
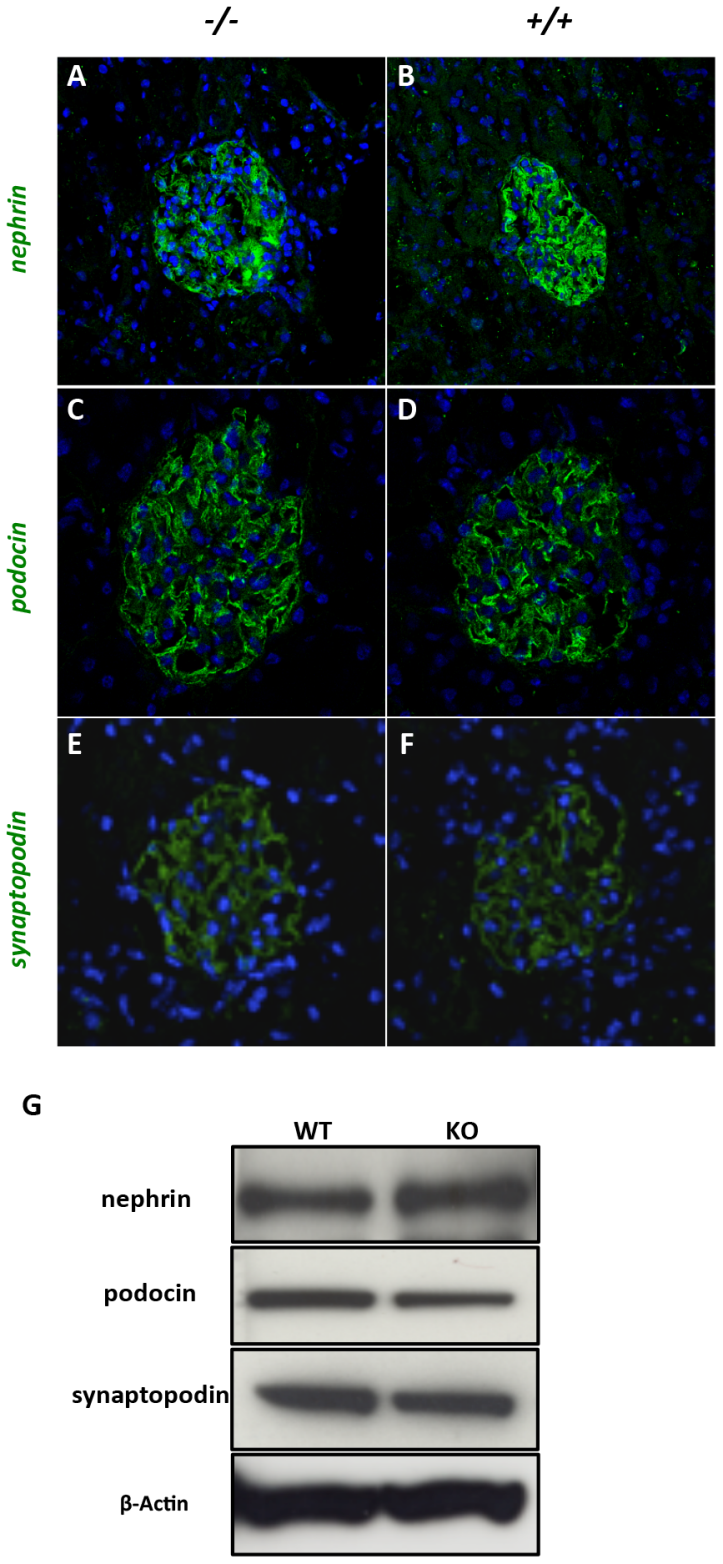
The expression of podocyte proteins in dendrin-deficient glomeruli. Slit diaphragm proteins nephrin (a–b) and podocin (c–d) are observed as linear line around capillary loops in control (+/+) and knockout (−/−) mice. (e–f) The expressioin of foot process protein synaptopodin is unchanged in dendrin knockout glomeruli. DAPI staining (blue) showing nuclei. Magnifications: ×200. (g) Western blotting for nephrin, podocin and synaptopodin do not show any significant expressional difference between wildtype (wt) and knockout (ko) glomeruli. β-Actin, used as a loading control, shows similar expression levels in both fractions.

To investigate how the lack of dendrin in podocytes affects the gene expression in the glomerulus, we microarray-profiled glomeruli from dendrin knockout mice. As expected, the dendrin expression was downregulated significantly (≈400 fold) in knockout glomeruli. Besides the downregulation of dendrin, the expression profiles showed only rather little changes. A total of 108 probe sets were differentially expressed in the dendrin-deficient glomerulus, when fold-change (KO/WT) over ±1.5 times and individual raw p value<0.05 were used as filtering criteria. When applying a multiple test correction for the 45101 probe sets on the array, none of the corrected p value is under 0.05. Of these 108 probe sets, 63 probe sets were downregulated and 45 probe sets upregulated, with the maximal fold change being 3.5 fold (except dendrin, table S1). No significant expressional changes were detected in well-known podocyte genes, including slit diaphragm genes that have been shown to bind to dendrin. This microarray data set has been submitted to Gene Expression Omnibus database at NCBI (acc = GSE7676). Furthermore, immunostaining patterns for slit diaphragm proteins nephrin and podocin, as well as foot process protein synaptopodin were not altered in dendrin-deficient glomeruli in comparison to controls (Fig. 3). In line with this, protein expression levels of these nephrin, podocin and synaptopodin were not significantly changed in knockout glomeruli (Fig. 3G).

### Dendrin does not modulate response to LPS or BSA-overload induced kidney injury

**Figure 4:**
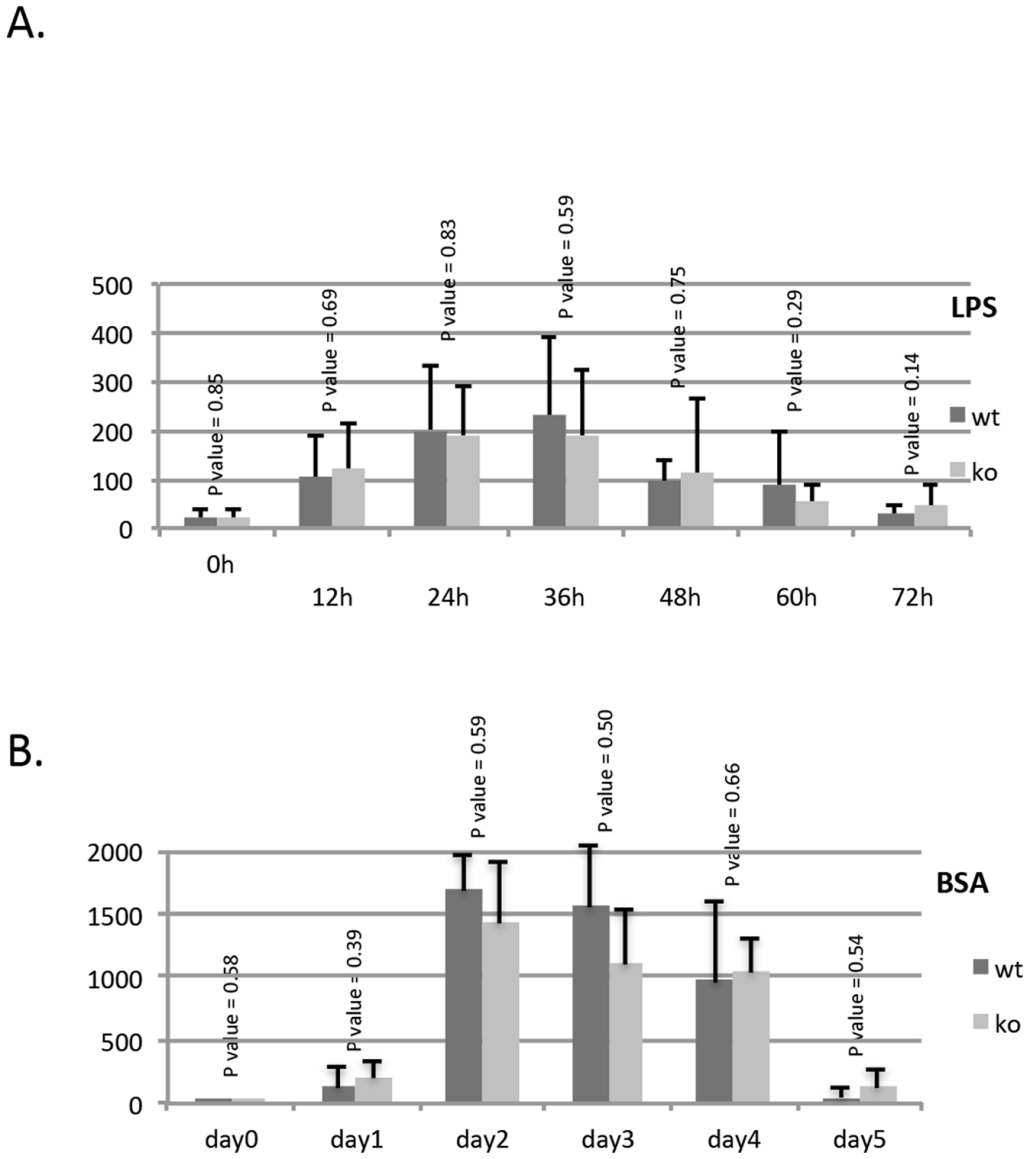
Albuminuria in LPS-nephropathy and BSA-overload models. (a) LPS-injection induce significant albuminuria in both dendrin knockout and control mice. Urinary albumin levels were similar in both mice (8 mice in each group). (b) BSA overload caused massive albuminuria in both dendrin-deficient and control mice. However, no significant differences were observed between the two groups (6 knockout and 5 control mice were included). Units in the Y axes indicate albuminuria level as analyzed by Quantity One software.

To study if dendrin has a role in compensatory mechanisms in the glomerulus, we challenged dendrin knockout mice with LPS-injection and BSA overload. These two models are well-established and mimic pathological conditions in the kidney [17]. Both LPS-injection and BSA overload resulted in a significant albuminuria (Fig. 4a–b). However, no significant difference was observed between the dendrin-deficient and control mice (Fig. 4a–b). Thus, dendrin does not seem to affect the response to glomerular injury in LPS-induced and BSA overload mouse models.

### Wtip and Gadd45a bind to dendrin

To analyze protein-protein interactions of dendrin, we performed a yeast two-hybrid screen. To identify meaningful interactions occurring in the podocyte, we used our own glomerular cDNA library [16] as a prey-library. The screening fished out 15 candidate proteins (Table 1). Two of the candidates caught our attention, Wtip and Gadd45a. Wtip has been shown to localize to the slit diaphragm and shuttle to nucleus in injured podocytes [18], similarly to the previous report for dendrin [10]. Gadd45a was, on the other hand, significantly upregulated in the dendrin-deficient glomeruli (table S1). Gadd45a encodes a protein involved in cell cycle regulation, DNA repair and genomic stability [19].

**Table 1:** Positive clones in yeast two-hybrid assay using dendrin as a bait.

Heterogeneous nuclear ribonucleoprotein F (hnrpf)
Mitochondrial ribosomal protein L38 (mrpl38)
Coiled-coil domain-containing protein 80 (Ccdc80)
Polymerase (RNA) III (DNA directed) polypeptide
General transcription factor II I repeat
Zinc finger and BTB domain containing 17
DnaJ (Hsp40) homolog, subfamily C, member 14
Integrin beta 4 isoform 2
Growth arrest and DNA-damage-inducible 45 alpha (gadd45a)
Filamin alpha
Calpain small subunit 1
Bone morphogenetic protein 4 precursor (BMP-4)
WT1-interacting protein (wtip)
Protein containing single MORN motif in testis(morn2)
Octamer-binding transcription factor 3 (Oct-3)

**Figure 5:**
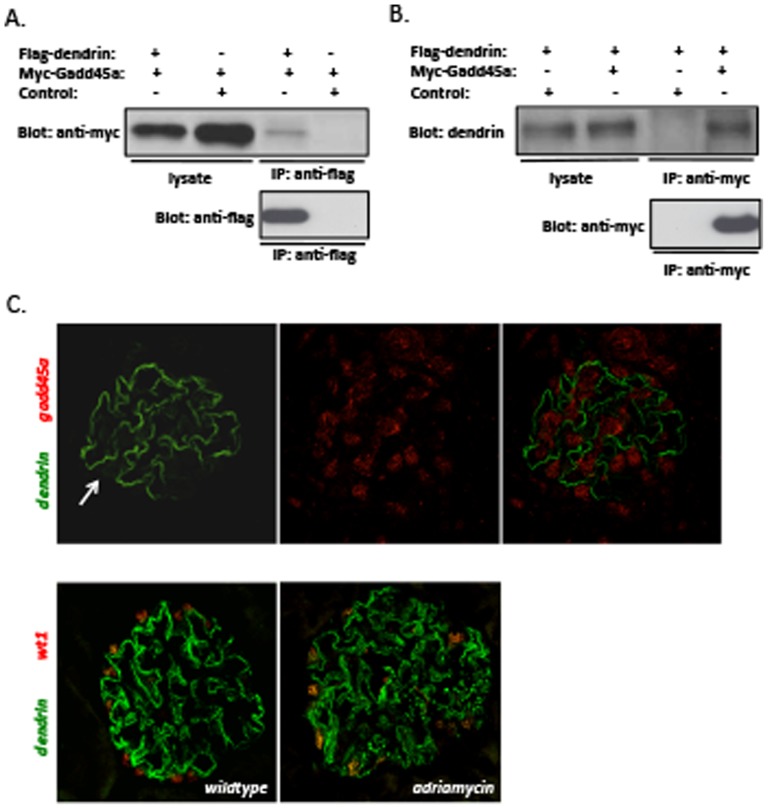
Analysis of interaction of dendrin with Gadd45a. (**a**) Immunoprecipitation (IP) of double transfected cells with anti-flag antibody brings down myc-tagged Gadd45a (Myc-Gadd45a) together with flag-tagged dendrin (Flag-dendrin). Control experiments do not bring down Myc-Gadd45a. (**b**) IP with anti-myc antibody co-immunoprecipitates flag-dendrin, whereas the control IP with a non-related expression construct does not bring down flag-dendrin. Input lysates from these experiments show expression of recombinant proteins. (**c**) Double immunofluorescence staining shows partial colocalization of dendrin and Gadd45a in podocyte nuclei. The positivity for dendrin in podocyte nuclei is increased in the adriamycin-induced nephropathy model. Podocyte nuclei are counterstained with wt1. Magnifications: ×200.

The assocation of dendrin with Gadd45a was confirmed by transfecting HEK293 cells with flag-tagged dendrin (f-dendrin) and myc-tagged Gadd45a (m-Gadd45a) constructs, followed by co-immunoprecipitation. Immunoprecipitation of f-dendrin with anti-flag antibody co-immunoprecipitated m-Gadd45a in double-transfected cells, whereas no m-Gadd45a was detected in the control immunoprecipitation (Figure 5a). Conversely, immunoprecipitation with anti-myc antibody brought down f-dendrin, whereas control immunoprecipitation experiments did not co-immunoprecipitate f-dendrin (Figure 5b). The analysis of cell lysates showed expression of recombinant proteins in both experiments (Figure 5a–b). To validate these results, we analyzed the localization of dendrin and Gadd45a in the glomerulus. In the normal glomerulus, strong immunoreactivity for dendrin was observed as a linear line around capillary loops indicating localization to foot processes (Fig. 1b). In addtion, a weak reactivity in nuclei in 62% (82/133 in 10 glomeruli) of podocytes (Fig. 5c) was observed. No dendrin was detected in nuclei of other glomerular cells. Staining for Gadd45a was detected in nuclei of all glomerular cells, including podocytes (Fig. 5c). Importantly, the staining for dendrin in podocyte nuclei overlapped with that of Gadd45a. In adriamycin-induced nephropathy (supporting fig. S2), immunoreactivity for dendrin in nucleus was increased as 95% (124/133 in 10 glomeruli) of podocyte nuclei showed reactivity for dendrin (doublestained with podocyte marker wt1, Fig. 5c).

**Figure 6:**
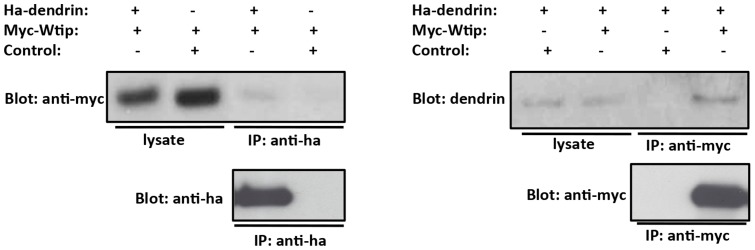
Analysis of interaction of dendrin with Wtip. (**a**) Immunoprecipitation (IP) of double transfected cells with anti-ha antibody brings down myc-tagged Wtip (Myc-Wtip) together with ha-tagged dendrin (ha-dendrin). Control experiments do not bring down Myc-Wtip (**b**) IP with anti-myc antibody co-immunoprecipitates ha-dendrin, whereas the control IP with a non-related expression construct does not bring down ha-dendrin. Input lysates from these experiments show expression of recombinant proteins.

We confirmed the interaction between dendrin and Wtip using co-immunoprecipitation experiments. We double transfected HEK 293 cells with ha-tagged dendrin (h-dendrin) and myc-tagged Wtip (m-Wtip). As controls, we used irrelevant expression constructs. Immunoprecipitation of h-dendrin with anti-ha antibody co-immunoprecipitated m-Wtip in double-transfected cells, whereas m-Wtip was not detected in the control immunoprecipitation (Figure 6a). Conversely, anti-myc antibody co-immunoprecipitated h-dendrin, whereas the control immunoprecipitation did not bring down h-dendrin (Figure 6b). The analysis of cell lysates showed expression of recombinant proteins in both experiments (Figure 6a–b). To validate these results, we tried to perform immunostaining experiments with various Wtip antibodies. However, in our hands, no reliable signal for Wtip was detected in immunohistochemical experiments and thus no colocalization studies could be made with dendrin.

## Discussion

The podocyte slit diaphragm interconnecting adjacent foot processes is probably the most critical part of the renal ultrafiltration barrier [20]. Dendrin is located at the cytoplasmic face of the slit diaphragm in where it binds to crucial slit diaphragm proteins nephrin and Cda2p [8], [10]. This, together with the fact that dendrin is highly specific for the podocyte slit diaphragm, prompted us to investigate the role of dendrin in the glomerulus by generating a dendrin knockout mouse line. Somewhat surprisingly, dendrin-deficient mice exhibited normal renal ultrafiltration barrier morphology and function, indicatíng that dendrin is dispensable for the normal development and function of the glomerulus filtration barrier. Next, we challenged dendrin deficient mice with LPS and BSA overload that are known to cause glomerular filtration barrier injury and proteinuria. Even under these pathological stimuli, we did not detect differences between dendrin-deficient and littermate control mice. Previously, dendrin has been reported to facilitate tgf-b-mediated pro-apoptotic signaling via its nuclear translocation in podocytes. Our results suggest that dendrin-mediated signaling does not play an important role in LPS and BSA-induced podocyte injuries.

Dendrin does not have any homologous proteins that can be expected to compensate for its loss. Therefore, to investigate possible compensatory changes in the glomerulus, we microarray-profiled dendrin-deficient glomeruli. We found relatively little expressional changes in the glomerulus, and notably, no significant changes in the expression of other slit diaphragm components. One of the genes upregulated in the dendrin-null glomerulus, Gadd45a, was interestingly identified in the yeast two-hybrid screen to bind dendrin. The interaction between dendrin and Gadd45a was confirmed with coimmunoprecipittion and colocalization experiments. Gadd45a encodes a protein involved in cell cycle regulation, DNA repair and genomic stability [19]. It is usually induced under stress condition, and arrests cell cycle at G2/M checkpoint and promotes apoptosis of damaged cells. In the kidney, it has been shown to modulate apoptosis in mesangial cells [21]. As dendrin has been shown to relocate to nucleus in injured podocytes and promote apoptosis, we think that Gadd45a may be in this same pathway and modulate dendrin-mediated apoptotic signals in podocytes.

Our interaction studies indicated that dendrin interacts with Wtip, a cytoplasmic component of the slit diaphragm. The Wtip-dendrin interaction is supported by previous studies that have shown that Wtip, similarly to dendrin, can shuttle to nucleus in injured podocytes [18]. It is unclear where dendrin and Wtip form a complex, in the slit diaphragm, nucleus, or both. In the nucleus, dendrin was recently shown to act as a transcription factor to promote the expression of cathepsin L [22]. Cathepsin L can proteolytically cleave the regulatory GTPase dynamin and the actin-associated adapter synaptopodin, and in that way cause a reorganization of the podocyte microfilament system resulting in proteinuria. As dendrin-deficient mice exhibit normal podocyte morphology and do not have proteinuria, it seems that there are also other mechanisms modulating cathepsin L mediated organization of the podocyte cytoskeleton.

In conclusion, our study indicates that dendrin is not needed for the normal development or function of the glomerular filtration barrier. Furthermore, our interaction studies showing that dendrin binds to Wtip and Gadd45a, support the idea that dendrin can act as a nuclear protein mediating transcription during glomerular injury.

## Supporting Information

Figure S1 **Characterization of anti-dendrin antibodies.** (**a**) All three anti-dendrin antibodies recognized a band around 80 kD in HEK293 cells transfected with full length mouse dendrin expression construct. No band was detected in control (nephrin) transfected cells. Tubulin was detected as a loading control. (**b**) In Western blotting of glomerular lysates, all three antibodies recognized a protein around 80 kD in both wildtype and knockout fractions. In Western blotting of brain and liver lysates, two of the antibodies recognized a band around 80 kD and another one around 88 kD. Tubulin was detected as a loading control.
(TIF)

Figure S2 **Characterization of Adriamycin nephropathy model.** Mice injected with Adriamycin develop massive albuminuria as observed by the analysi of urine from these mice collected 0, 4, 7 and 14 days after the injection. Two microliter of urine was loaded on SDS-page gel.
(TIFF)

Table S1 (XLS)
