# Supplementary figures and images for: Wtip- and Gadd45a-Interacting Protein Dendrin Is Not Crucial for the Development or Maintenance of the Glomerular Filtration Barrier
Source: PLoS One. 2013 Dec 20;8(12):e83133. doi: 10.1371/journal.pone.0083133 (PMC3869763; doi:10.1371/journal.pone.0083133)

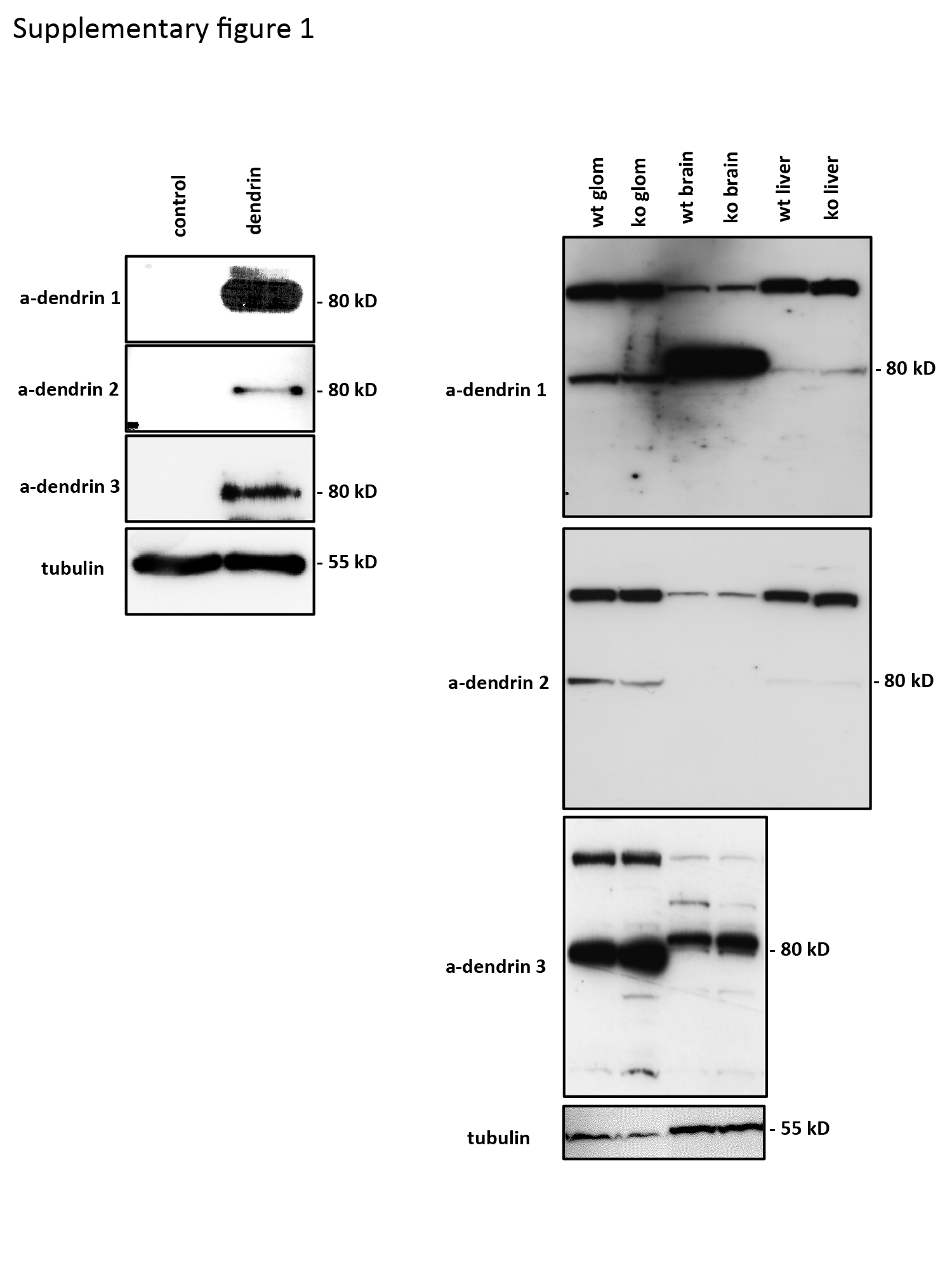

Supplement: Figure S1 — Characterization of anti-dendrin antibodies. (a) All three anti-dendrin antibodies recognized a band around 80 kD in HEK293 cells transfected with full length mouse dendrin expression construct. No band was detected in control (nephrin) transfected cells. Tubulin was detected as a loading control. (b) In Western blotting of glomerular lysates, all three antibodies recognized a protein around 80 kD in both wildtype and knockout fractions. In Western blotting of brain and liver lysates, two of the antibodies recognized a band around 80 kD and another one around 88 kD. Tubulin was detected as a loading control. (TIF) [file pone.0083133.s001.tif]

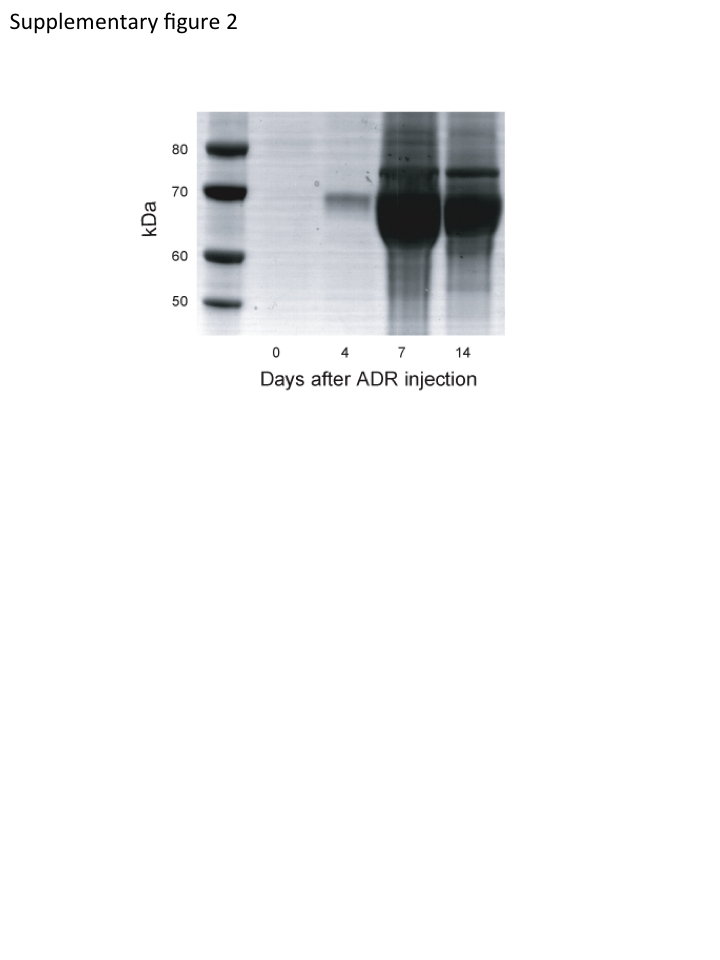

Supplement: Figure S2 — Characterization of Adriamycin nephropathy model. Mice injected with Adriamycin develop massive albuminuria as observed by the analysi of urine from these mice collected 0, 4, 7 and 14 days after the injection. Two microliter of urine was loaded on SDS-page gel. (TIFF) [file pone.0083133.s002.tiff]
